# Deciphering the Plastomic Code of Chinese Hog-Peanut (*Amphicarpaea edgeworthii* Benth., Leguminosae): Comparative Genomics and Evolutionary Insights within the Phaseoleae Tribe

**DOI:** 10.3390/genes15010088

**Published:** 2024-01-11

**Authors:** Yi-Nan Xiang, Xiao-Qun Wang, Lu-Lu Ding, Xin-Yu Bai, Yu-Qing Feng, Zhe-Chen Qi, Yong-Tao Sun, Xiao-Ling Yan

**Affiliations:** 1 Zhejiang Province Key Laboratory of Plant Secondary Metabolism and Regulation, College of Life Sciences and Medicine, Zhejiang Sci-Tech University, Hangzhou 310018, China; yinanxiang9@outlook.com (Y.-N.X.); llding6@163.com (L.-L.D.); fyq071008@163.com (Y.-Q.F.); 2 Eastern China Conservation Centre for Wild Endangered Plant Resources, Shanghai Chenshan Botanical Garden, Shanghai 201602, China; 3 East China Survey and Planning Institute, The National Forestry and Grassland Administration, Hangzhou 310019, China; sunyongtao329@sina.com

**Keywords:** chloroplast genomics, evolutionary biology, glycininae, Leguminosae phylogeny, phylogenetic relationships, plastome characterization, subtribal classification, systematics

## Abstract

The classification and phylogenetic relationships within the Phaseoleae tribe (Leguminosae) have consistently posed challenges to botanists. This study addresses these taxonomic intricacies, with a specific focus on the Glycininae subtribe, by conducting a comprehensive analysis of the highly conserved plastome in *Amphicarpaea edgeworthii* Benth., a critical species within this subtribe. Through meticulous genomic sequencing, we identified a plastome size of 148,650 bp, composed of 128 genes, including 84 protein-coding genes, 36 tRNA genes, and 8 rRNA genes. Comparative genomic analysis across seven Glycininae species illuminated a universally conserved circular and quadripartite structure, with nine genes exhibiting notable nucleotide diversity, signifying a remarkable genomic variability. Phylogenetic reconstruction of 35 Phaseoleae species underscores the affinity of *Amphicarpaea* with *Glycine*, placing *Apios* as a sister lineage to all other Phaseoleae species, excluding Clitorinae and Diocleinae subtribes. Intriguingly, *Apios*, *Butea*, *Erythrina*, and *Spatholobus*, traditionally clumped together in the Erythrininae subtribe, display paraphyletic divergence, thereby contesting their taxonomic coherence. The pronounced structural differences in the quadripartite boundary genes among taxa with unresolved subtribal affiliations demand a reevaluation of Erythrininae’s taxonomic classification, potentially refining the phylogenetic contours of the tribe.

## 1. Introduction

Leguminous plants, a large and economically significant family that have a pivotal role in agricultural systems as food, feed, and biofertilizers, present a compelling model for genetic and evolutionary studies [1,2,3]. The distinct symbiotic nitrogen-fixing capabilities of legumes underscore their ecological significance and offer a window into plant–microbe interactions and biogeochemical processes [4,5,6]. However, despite the significance of legumes, foundational taxonomic research within the family remains fraught with unresolved issues [7,8,9].

The Phaseoleae tribe belonging to the subfamily Papilionaceae comprises seven subtribes with eighty-four genera, which are classified under the unranked non-protein amino acid-accumulating clade (NPAAA clade) [10,11]. Yet, its subtribe classification remains elusive, with varying nomenclature across systems. J. Lackey’s chemotaxonomic revision following Bentham’s schema in the ‘Genera Plantarum’ presents a dichotomy in the distribution of canavanine among subtribes, proposing a seven-subtribe structure: Cajaninae, Kennediinae, Diocleinae, Phaseolinae, Ophrestiinae, Glycininae, and Erythrininae [12]. The USDA’s Germplasm Resources Information Network (GRIN) recognizes the absence of Erythrininae and the addition of Clitoriinae. Within this framework, certain taxa remain unassigned to any subtribe. Flora Reipublicae Popularis Sinicae (FRPS) offers yet another perspective, placing genera, like *Apios*, *Butea*, *Cochlianthus*, *Erythrina*, *Mucuna*, *Spatholobus*, among others, into the subtribe Erythrininae based on morphological characteristics [13], whereas these genera are categorized within undetermined subtribes in the GRIN. This taxonomic ambiguity underscores the imperative for refined investigations to elucidate the phylogenetic intricacies within the Phaseoleae tribe. Similarly uncertain is the tribe’s position within the Leguminosae family, notably its alleged association with the Desmodieae and Psoraleeae tribes in the Indigoferoid/Millettioid clade [9,14]. These insights suggest that Phaseoleae’s classification is intermingled with other tribes, like Millettieae and Abreae, indicating a dispersed phylogenetic identity lacking clear delineation. A deeper understanding of the molecular characteristics of each subtribe will enhance the classification of Phaseoleae and provide more information for phylogenetic reconstruction.

*Amphicarpaea edgeworthii* is an annual widely distributed species that attracts considerable attention due to its three types of flowers (fruits), subterranean cleistogamous, aerial cleistogamous, and aerial chasmogamous, and serves as a model plant for studying complex flowering patterns and reproductive strategy [15,16,17,18]. It grows in the pool of the forests and geographic areas of grasslands in mountainous areas. In recent years, there has been an in-depth exploration of the nuclear genome of *A. edgeworthii* [19,20,21]. However, its chloroplast genome has yet to be fully analyzed, and there are unresolved relationships within the subtribe Glycininae. In a previous phylogenetic study of *A. ferruginea*, the genera *Amphicarpaea* and *Pueraria* were identified as sister taxa, forming a polyphyletic relationship with the genera *Glycine* and *Mucuna*, and *Glycine* and *Spatholobus* were clustered as clades [22]. This contradicts the existing classification system. *Amphicarpaea* and *Glycine* are classified under the Glycininae subtribe, while *Spatholobus* and *Mucuna*, as previously mentioned, are placed outside, and the subtribal allocation of these taxa is still incompletely defined. In a tree constructed using the *matK* gene, *Amphicarpaea*, *Pueraria*, and *Glycine* within the Glycininae subtribe exhibit a polytomy relationship [8]. In another RPS16 intron sequence tree, *Amphicarpaea*, *Glycine*, *Pueraria*, and *Teramnus* form three subclades, while *Amphicarpaea* is sister to a clade consisting of *Glycine* and *Teramnus*, and it is noteworthy that *Pueraria* is not monophyletic internally [23]. The Glycininae subtribe holds substantial economic importance and prominence. Among its most important members is soybean (*Glycine max*), whose seeds are rich in protein and serve as raw materials for various soy products and oil extraction. They can be utilized in the production of health supplements and pharmaceuticals [24,25]. Additionally, these plants exhibit robust nitrogen-fixing capabilities, contributing to soil improvement and promoting sustainable agricultural development [26]. Therefore, analyzing the plastome of this subtribe, determining its phylogenetic relationships with disputed genera, and elucidating its position within the Phaseoleae tribe are meaningful endeavors.

The progress of high-throughput sequencing technologies in the past two decades has enhanced the efficiency and quality of plastid genome sequencing. The plastome is a vital genomic region in plants, characterized by maternal inheritance and circular DNA structure [27,28]. It contains several essential genes involved in critical biological processes, such as photosynthesis, plastid protein import, fatty acid biosynthesis, and proteolysis [29,30,31]. The plastome has proved to be a valuable tool not only for establishing plant phylogenetic relationships, developing DNA barcodes, and creating molecular markers but also for studying regulatory mechanisms of photosynthesis [32,33,34,35,36,37]. Currently, the National Center for Biotechnology Information (NCBI) website has published over 1300 complete plastomes within the legume family, spanning more than 350 species. The species with the highest number of sequence records are *Medicago minima*, *Pueraria montana*, and *Trifolium pratense*, all belonging to the Papilionoideae subfamily. These complete chloroplast datasets have been applied to explore phylogenetic relationships at various scales within the Leguminosae family, including the family level (Leguminosae), subfamily level (Papilionoideae), evolutionary branch level (Millettioid/Phaseoloid clade), and genus and subgenus [14,38,39,40,41]. Previous studies did not sample and discuss the tribe and subtribe levels within the unresolved Millettioid/Phaseoloid clade, particularly focusing on the ambiguous boundaries of Phaseoleae [14].

Here, we generated the latest complete plastome of *A. edgeworthii* and conducted the first analysis of its architecture in comparison with six other species within Glycininae. We performed a phylogenetic analysis of a total of thirty-five species within Papilionaceae, including seven subtribes and five genera awaiting subtribal assignment, and discussed the distribution patterns of boundary genes among different subtribes and provided recommendations for the internal classification of Phaseoleae.

## 2. Results and Discussion

### 2.1. The Overall Structure and General Features of the A. edgeworthii Chloroplast Genome

**Figure 1:**
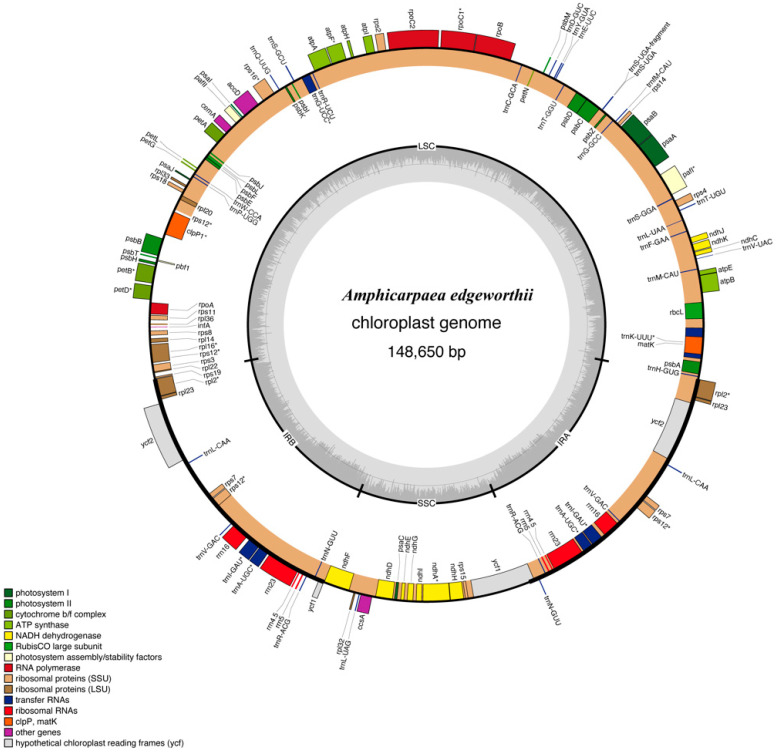
The structure of the *A. edgeworthii* plastome. Transcription occurs clockwise for genes located inside the circles and counterclockwise for those located outside the circles. The functional genes are indicated by colorful bars. The GC and AT contents of the inner circle are denoted by the dark gray and light gray colors, respectively. Genes with an asterisk * indicate the presence of introns.

The plastome of *A. edgeworthii* has a quadripartite, circular topology with a length of 148,650 base pairs (bp) (Figure 1). The plastome consists of a pair of inverted repeats (IRb and IRa), each 23,504 bp, a Small Single-Copy (SSC) region of 17,967 bp, and a Large Single-Copy (LSC) region of 83,675 bp. A total of 49,351 bp make up the genome’s non-coding region, which comprises introns and intergenic spacers, while the remaining 75,693 bp are coding (CDS). The GC content of the LSC and SSC regions is 32.9% and 28.7%, respectively, whereas in the inverted repeats IRa and IRb, it is 42.3% for both. Thus, IRs have a larger proportion of GC than the SSC and LSC regions (Table 1). The GC percentage at the first, second, and third positions in the CDS sequence are 44.43%, 36.74%, and 26.69%, respectively.

The annotation of *A. edgeworthii* plastome revealed a total of 128 genes (84 protein-coding genes, 36 tRNAs genes, and eight rRNAs genes); 86 genes are present in the LSC (66 protein-coding genes and 20 tRNA genes) and 12 genes are present in the SSC (11 protein-coding genes and one tRNA), while the remaining 14 genes (six tRNAs, four rRNAs, and four protein-coding genes) are in the IRa and IRb regions (Table 2).

**Figure 2:**
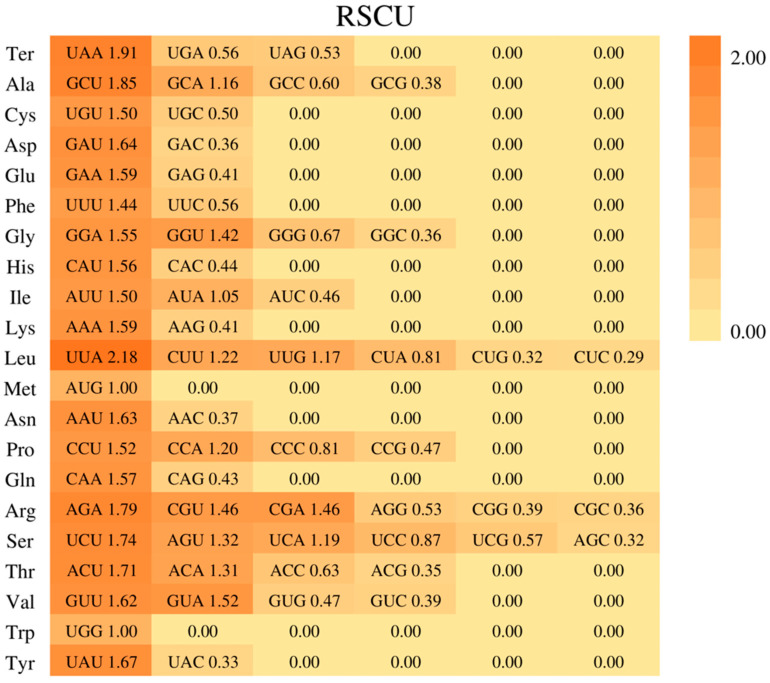
A heatmap of the RSCU values in the *A. edgeworthii* plastome.

**Figure 3:**
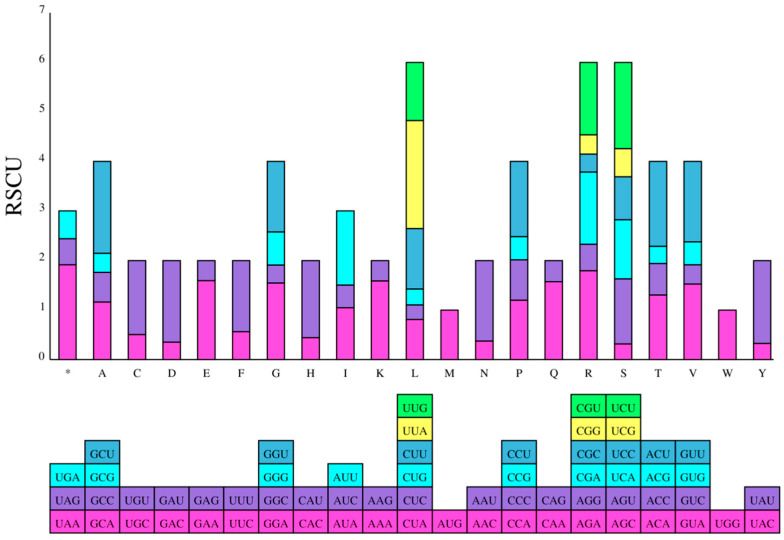
The stacked bar chart of RSCU values, with amino acids on the *x*-axis and the frequency of each codon on the *y*-axis. For each amino acid (column), represent each codon encoding it with a specific color. The * columns denote stop codons.

The result of relatively synonymous codon usage (RSCU) was that 64 codons were employed (Figure 2 and Figure 3). Of these, 31 codons have RSCU values lower than 1, and 31 codons have RSCU values higher than 1. A total of 96.8% of codons with high RSCU values have Cytosine (C) or Guanine (G) endings, and 93.5% of codons with lower RSCU values have Thymine (T) or Adenine (A) endings. This pattern of the third codon usage was also observed in other species of legumes [42,43]. AUG and UGG are codons without bias (i.e., with RSCU values = 1), while the termination codon, UAA, has a value of 1.9125.

In the plastome of *A. edgeworthii*, there are coding genes that contain introns (Table 3). Introns are reported to exist in some of the protein-coding and tRNAs genes of the plastome other angiosperms. Out of the 128 coding genes, 19 are characterized by one or two introns. Of these nineteen genes, six are tRNAs and thirteen are protein-coding genes. 

Ten of the intron-containing genes are located in the LSC, one gene is in the SSC, and the remaining four are in the inverted repeat regions. ATP-dependent protease subunit p gene (*clpP1*) and photosystem I-related gene (*pafI*) possess two introns, while the remaining seventeen genes have only one. The tRNA gene, *trnK-UUU*, is the gene with the longest intron due to the inclusion of *matK* within its sequence.

### 2.2. Repeat Analyses

**Figure 4:**
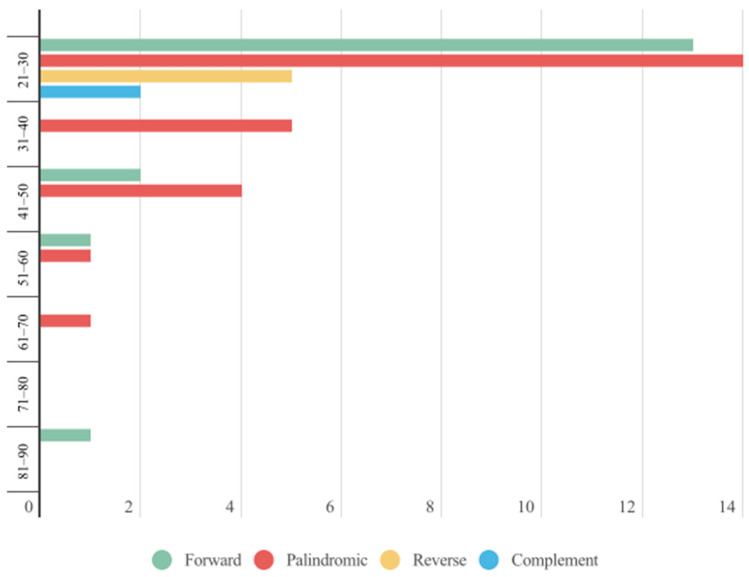
Bar chart showing the distribution of four scattered repetitive sequences across length intervals in the plastome of *A. edgeworthii*.

A comprehensive statistical analysis was conducted on the dispersed repetitive sequences in the plastome of *A. edgeworthii*. The analysis identified four types of dispersed repeat sequences with lengths greater than 20 bp, namely forward (F), palindromic (P), reverse (R), and complement (C) (Figure 4). Notably, P-type repeats were the most frequent, with a total count of 24. Interestingly, R and C types were only detected within the length range of 21–30 bp, while F-type repeats were exclusively identified within the length range of 81–90 bp, suggesting that they may play a unique role in the structural and functional organization of the plastid genome. These findings offer new insights into the nature and distribution of dispersed repeats in the plastome of *A. edgeworthii* and their potential impact on plastome evolution and function. Specifically, these dispersed repeats could induce DNA recombination, mutation, and gene transfer, ultimately contributing to the complexity and diversity of plastomes.

SSRs, also known as microsatellites, are valuable genetic markers for various applications in plant and animal breeding, conservation biology, and population genetics. Analyzing the distribution and diversity of SSRs in the plastome can lead to the development of SSR markers capable of distinguishing between different plant populations, species, or varieties based on their unique genetic fingerprints. SSR sequences in the plastomes of *A. edgeworthii* were identified using the MISA program. In this study, a total of 79 microsatellites were discovered. Mononucleotides were the most frequent SSRs, comprising approximately 59.49% of the total SSRs, with the majority being composed of A/T. Among dinucleotides, only AT/AT was found, while trinucleotides were represented by AAG/CTT and AAT/ATT. The tetranucleotides included AAAG/CTTT, AAAT/ATTT, AATC/ATTG, AATT/AATT, and AGAT/ATCT. No pentanucleotides or hexanucleotides were discovered. In terms of quantity, SSRs are mainly distributed in the LSC and SSC regions of the plastome. The LSC region harbors the most diverse types of SSRs, including mononucleotides, dinucleotides, trinucleotides, and tetranucleotides. Each of the IR regions contains two mononucleotides. The SSC region includes ten mononucleotides and one tetranucleotide.

**Figure 5:**
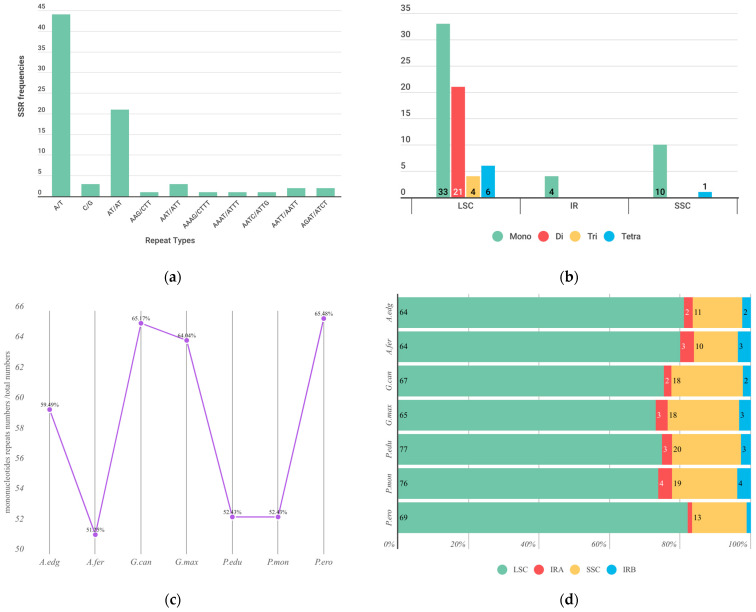
Single sequence repeat (SSR) analysis of the plastome in Glicininae. (**a**) Frequency of different SSR motifs in different repeat types in *A. edgeworthii* plastome; (**b**) distribution of SSR in LSC, SSC, and IR regions; (**c**) proportion of single-nucleotide SSRs in seven Glycininae species; (**d**) proportion of single-nucleotide SSRs in seven Glycininae species.

We also conducted SSR analysis on other species within Glycininae (Figure 5). Mononucleotides accounted for the highest proportion among the SSRs of seven species. *Glycine canescens* had the highest proportion of mononucleotides, followed by *Pachyrhizus erosus* and *G. max*, with all three species having mononucleotide ratios of around 65%. The lowest was observed in *A. ferruginea*, at 51%. The distribution pattern of SSRs was similar among the seven species, mainly located in the LSC region, with the SSRs in *P. erosus*’s LSC region accounting for the highest proportion, exceeding 80%.

The telomer restriction fragment (TRF) analysis report indicates the presence of satellite DNA and minisatellite DNA, excluding SSRs ranging from 1–6 bp in length. Tandem repeat sequences in the plastomes were identified using the program TRF (percent matches ≥ 95%, score > 90). This analysis revealed 24 tandem repeats. Overall, the period size ranged between 10 and 27 bp, and the number of copies aligned with the consensus pattern was between 1.9 and 4.1. The highest number of occurrences was observed for a period size of 27 bp, followed by 14 bp.

### 2.3. Comparative Analysis of the Plastome in Subtribe Glycininae Species

To evaluate the level of plastomes divergence in Glycininae, the newly sequenced plastome *A. edgeworthii* was compared with plastomes from six other Glycininae species. 

**Figure 6:**
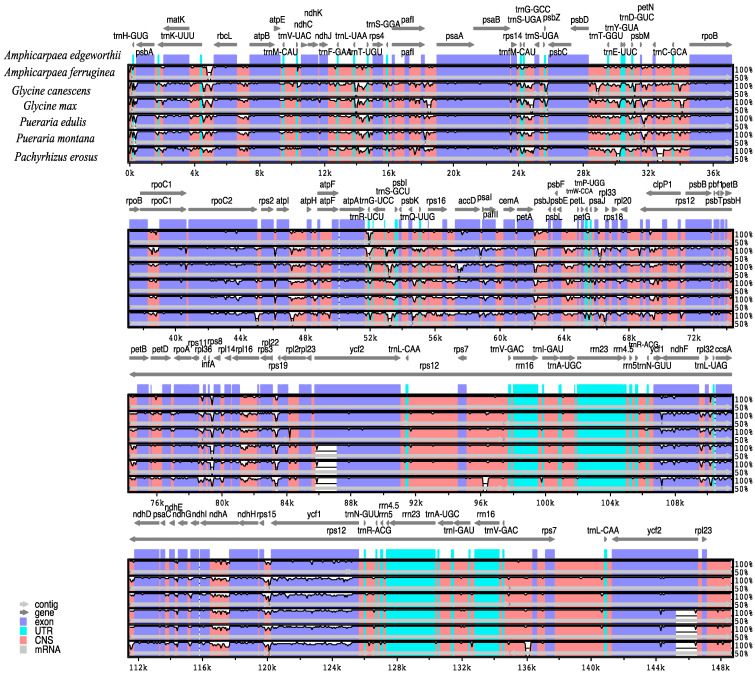
Variable regions in the plastome of seven Glycininae species. The top arrow represents the direction of transcription; the blue and pink colors denote protein-coding and conserved non-coding sequences, respectively; light green denotes tRNAs and rRNAs. The plastome coordinates are shown on the *x*-axis, and the percentage identity ranges from 50% to 100% on the *y*-axis.

**Figure 7:**
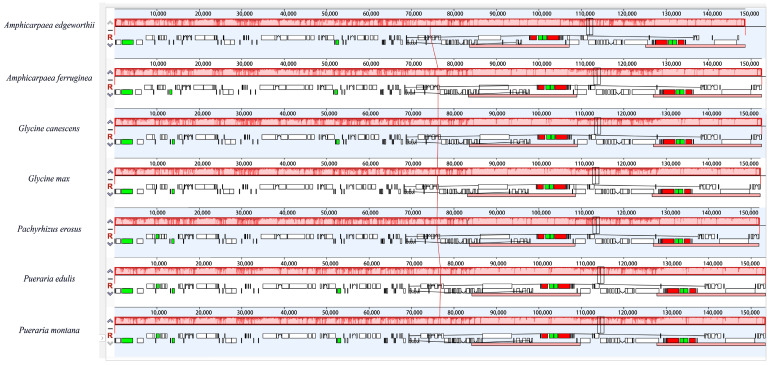
Plastome alignment visualized using a Mauve multiple alignment plot. The color of each sequence line represents its position in the genome sequence, with similar regions having similar colors. The color boxes display the annotated features of the plastome sequences, where green corresponds to tRNAs, red corresponds to rRNAs, and white represents genes containing CDS.

The plastomes were aligned and analyzed using mVISTA to investigate the conservation of different regions (Figure 6). The UTR region exhibited the highest level of conservation, followed by protein-coding regions and introns. Of the four areas analyzed, the IRa and IRb were found to be more conserved than the SSC and LSC. Variations in the sequences of certain genes, such as *rpoC2*, *rpoB*, and *rps3*, were observed, albeit to a small degree. Conversely, significant sequence divergence was detected in genes such as *matK*, *accD*, *pafII*, *ndhF*, and *ycf2*, which could potentially serve as barcodes for identifying and authenticating Justiceae species. Additionally, these regions may be valuable resources for inferring the phylogenetic relationships of Glycininae. The analysis using Mauve showed that the seven species within Glycininae exhibited a highly conserved linear arrangement with respect to both gene order and rearrangements (Figure 7).

**Figure 8:**
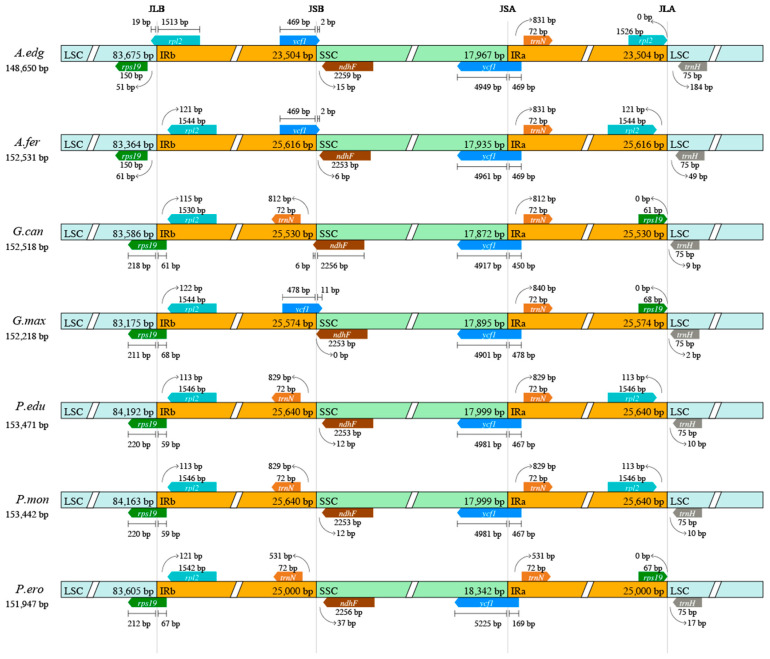
Structural variation in the junction of inverted repeat and single-copy regions among the seven plastomes of Glycininae. (JSA: junction of the SSC and the IRA; JLB: junction of the LSC and the IRB; JSB: junction of the SSC and the IRB).

We also compared the JLB, JSB, JSA, and JLA boundaries (Figure 8). The results showed some similarities and variations among the compared plastomes. The length of the seven plastomes ranged from 148,650 bp (*A. edgeworthii*) to 153,471 bp (*Pueraria edulis*). The boundaries of JSA, JLB, and JLA are very conservative, with the main differences being reflected in the boundary of JSB. In *A. edgeworthii*, *A. ferruginea,* and *G. max,* the *ycf1* gene spans from the IRb region to the SSC region, whilst in *G. canescens,* this position hosts the *ndhF* gene. In *P. edulis*, *P. montana*, and *P. erosus,* the *trnN* gene is located within the IRB region 500 to 800 bp away from the JSB region. Their boundaries show very small degrees of contraction and expansion, with differences between species not exceeding 300 bp. Overall, the plastome structure in the subtribe Glycininae appears to be stable and relatively homogeneous. The lack of large-scale genome rearrangements points to close evolutionary relationships between the subtribe species and a relatively recent origin of the clade.

### 2.4. Divergence of Protein-Coding Gene Sequences

**Figure 9:**
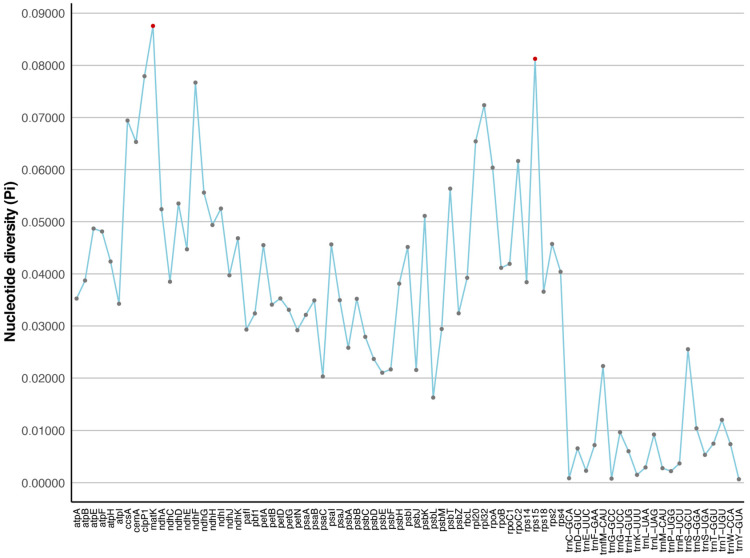
Sliding window analysis of nucleotide variability among the seven Glycininae species plastomes (window length: 600 bp; step size: 200 bp).

We performed manual curation on the annotated genome to eliminate any gene annotations that might have been duplicated. This work resulted in the identification of 79 single-copy orthologous genes from the genome sequences of 35 species. We calculated the level of nucleotide diversity (Pi) for each of these genes separately (Figure 9). The calculated pi values ranged from 0.00066 to 0.08757. Genes with high Pi values (>0.06) in the plastome include *matK*, *rps15*, *clpP1*, *ndhF*, *rpl32*, *ccsA*, *rpl20*, *cemA*, and *rpoC2*, with *matK* having the highest Pi value. Among these, *matK* encodes maturase, a protein that splices Group II introns, whereas *ndhF* is involved in the electron transfer chain of photosynthesis. Three other high-Pi genes code for ribosomal proteins (*rps15*, *rpl32*, *rpl20*), and the remaining ones also have significant functions. *ccsA* plays a role in plant response to oxidative stress, *cemA* is involved in the synthesis and maintenance of the cell wall, and *rpoC2* is a critical component of RNA polymerase. These genes may be associated with environmental changes, which are helpful for understanding the interaction between the Phaseoleae tribe and the environment during evolution. 

### 2.5. Phylogenetic Analysis

Past taxonomic disagreements have centered around whether the Erythrininae and Kennediinae should be considered independent subtribes within the subtribal classification system of Phaseoleae. Our study addressed this issue by analyzing a representative sample of the Phaseoleae tribe, comprising seven subtribes, including Erythrininae and Kennediinae.

**Figure 10:**
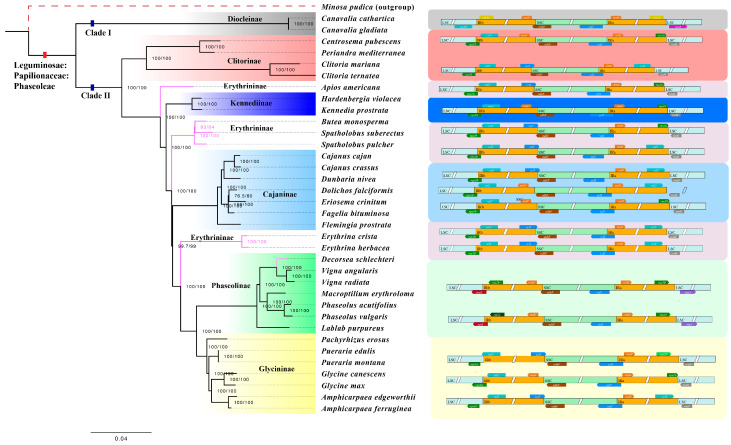
Phylogenetic tree of Phaseoleae reconstructed using the plastomes of 35 taxa. The branch nodes’ numbers represent the posterior probabilities values (PPs). Colors shaded on the branches indicate the corresponding subtribes. The pink branches on the phylogenetic tree represent members from the Erythrininae. On the right is the pattern of structural variations in the junctions of inverted repeat (IR) and single-copy (SC) regions for each subtribe, with genes at the IR/SC junctions indicated.

Our phylogenetic analysis yielded a highly resolved and well-supported evolutionary tree, clearly delineating the relationships among all the studied species (Figure 10). This tree comprises two primary sister clades: the Diocleinae clade (Clade I) and a clade consisting of the remaining species (Clade II). Within Clade II, the Erythrininae are polyphyletic, while the other five subtribes are monophyletic. The Glycininae clade, occupying a derived position, establishes a sister relationship with the Phaseolinae clade. Genes located at the junctions in Phaseoleae are *rps11*, *rps19*, *rps8*, *rps3*, *rpl2*, *rpl22*, *rpl23*, *trnN*, *ndhF,* and *ycf1*.

Clitorinae forms a sister relationship to the rest of the species in Clade II. The Kennediinae clade showed a relatively early diverged position compared to the remaining subtribes. Lackey once speculated on the synonymic between Kennediinae and Diocleinae, noting similarities in floral, pod, and seed attachment. However, the absence of bracteoles, the prominent aril, and geographical isolation supported the independence of Kennediinae [12]. The recent recognition of Kennediinae as an independent subtribe was mentioned in studies related to cotyledon areoles in 2008 [44]. Currently, phylogenetic evidence increasingly supports the classification of Kennediinae as a distinct subtribe within Phaseoleae.

Regarding the polyphyletic Erythrininae, species taxonomically ascribed to this subtribe, namely *Apios*, *Butea*, *Cochlianthus*, *Erythrina*, *Mucuna*, and *Spatholobus*, are separated into four subclades (pink clades, Figure 10). Three subclades of Erythrininae form a paraphyletic group with Kennediinae, Cajaninae, and Phaseolinae. It is noteworthy that *Decorsea schlechteri* belonging to Erythrininae are embedded within Phaseolinae, showing a sister relationship with *Vigna*. *Apios americana* shares specific genes on JLB and JSB with the genus *Clitoria*, while other genes on JLA and JSA are shared with the Kennediinae subtribe, reflecting its evolutionary intermediary position. For the genus *Spatholobus*, two species exhibit boundaries consistent with the *Cajanus* genus. In contrast, for the *Butea* genus, boundary genes at JLB and LSB align with *Cajanus*, whereas JLA and JSA boundary genes match with the Kennediinae subtribe. *Erythrina*, as the representative genus, shows that Asian species are derived from a primarily African clade, with South American species being basal [45]. By comparing the junctions, the *Erythrina* genus shares some boundary genes with the *Cajanus* genus. Lackey considered that Erythrininae could potentially include various lineages originating from a Galegeae–Dalbergieae stock [12]. Despite this, Galegeae is in the IRLC clade (Inverted Repeat-Lacking Clade), and Dalbergieae is in the Dalbergioid clade. We believe that the classification based on morphology in FRPS needs to be improved, and the incertae sedis in the GRIN also needs to be revised in the light of phylogenomics.

In addition to the lingering issue of the status of Kennediinae and Erythrininae, the position of *Decorsea*, an undetermined subtribe genus traditionally placed in subtribe Phaseolinae, which exhibits a sister relationship with the genus *Vigna* in our tree (Figure 10), is also noteworthy. Its boundary genes are also in line with those of the *Vigna* genus, suggesting the possibility of incorporating *Decorsea* into the Phaseolinae subtribe.

Within the Glycininae subtribe, *Amphicarpaea* and *Glycine* form a clade, which is sister to *Pueraria*. Our findings are similar to the tree constructed using *rps16* intron sequences by Lee et al. [23]. In their analysis, *Pueraria* exhibits non-monophyly, whereas we utilized complete genomes from two species within the *Pueraria* genus, and both remain non-dispersed.

## 3. Materials and Methods

### 3.1. Plant Material and DNA Extraction

Leaves from healthy individuals of *A. edgeworthii* were collected and immediately frozen in liquid nitrogen for preservation. Leaves were prepared for DNA extraction with care to avoid excess mucilage. The total genomic DNA was extracted using a TIANGEN Plant Genomic DNA kit (Beijing, China) following the manufacturer’s guide. Then, DNA concentration and quality were assessed by a NanoDrop 2000 spectrophotometer (Thermo Scientific, DE, USA) and 1% agarose gel electrophoresis. Qualified DNA was sent to Major-bio (Shanghai, China) for library preparation and high-throughput sequencing using Illumina Novaseq 6000 Platform (Illumina, CA, USA) with 150 bp paired-end reads.

### 3.2. DNA Sequencing and Genome Assembly

To prepare the DNA samples, 1.0 µg of high-quality genomic DNA was sheared into fragments of approximately 350 bp using a Covaris S220 instrument (Covaris, Woburn, MA, USA). The construction of sequencing libraries was performed using the NEBNext Ultra II DNA Library Prep Kit (New England Biolabs, Ipswich, MA, USA) following the manufacturer’s instructions. Subsequently, library quantification was conducted with a Qubit dsDNA HS Assay Kit (Life Technologies, Carlsbad, CA, USA), and size distribution was assessed using an Agilent 2100 Bioanalyzer (Agilent Technologies, Santa Clara, CA, USA). After pooling, the libraries were subjected to PCR enrichment and purified using the AMPure XP system (Beckman Coulter, Brea, CA, USA). The qualified libraries based on projected data volume and effective concentration were then sequenced on an Illumina HiSeq X Ten platform (Illumina, San Diego, CA, USA).

The raw sequencing data were processed using PRINSEQlite v0.20.4 to obtain high-quality reads (5.2 GB) by filtering out low-quality reads, adapters, and ambiguous bases [46]. Plastome assembly was performed using NOVOPlasty 4.2 software, which employs a de novo assembly approach with a reference seed for the plastome [47]. The seed sequence was obtained from a closely related species and was used as a reference for the assembly process. The high-quality reads were used to generate contigs with k-mer sizes of 39, and the contigs were then merged iteratively to produce a draft plastome assembly.

### 3.3. Genome Annotation: Genes and Repetitive Elements

Gene annotation was carried out with GeSeq (Annotation of Organellar Genomes) and CPGAVAS2 using *Medicago turbinate* (NC_068638.1) and *G. max* (CM010429.1) as reference genomes [48,49]. The 36 annotated plastomes were manually curated by reviewing gene boundaries and correcting misaligned gene fragments. Additionally, the orientation of the SSC and LSC regions of all genomes was standardized. A graphical representation of the plastome was drawn using OGDRAW software (http://ogdraw.mpimp-golm.mpg.de/, accessed on 25 March 2023) [50].

Simple sequence repeats (SSRs) were identified and analyzed using MISA (MIcroSAtellite identification) (https://webblast.ipk-gatersleben.de/misa/, accessed on 26 March 2023) [51]. For the detection of SSR motifs in mononucleotides, dinucleotides, trinucleotides, tetra-, penta-, and hexanucleotides, the minimum number of repeat units used were ten, five, four, and three, respectively. The online software REPuter was used to characterize the long repeat sequences in the plastome [52]. To identify dispersed repetitive elements, we employed a TRF (Tandem Repeats Finder) and performed the analysis with the default parameter [53].

### 3.4. Genome Comparison and Phylogenetic Analysis

For whole structure analysis, using the annotation of *A. edgeworthii* as a reference in the LAGAN mode, the plastomes of six other species of Glycininae (*A. ferruginea*, *G. max*, *G. canescens*, *P. montana*, *P. edulis*, *P. erosus*) were compared using the tool mVISTA [54]. The border regions of the manually curated plastomes were visualized on the online Genepioneer Cloud Platform (http://cloud.genepioneer.com:9929, accessed on 26 March 2023). To identify structural variations and sequence divergences between the genomes, we utilized the software progressiveMauve adopting automatic calculation of seed weights and the minimum LCB (locally collinear block) score parameter [55]. 

To analyze the phylogenetic relationships among 36 species, including 35 species of Phaseoleae and one outgroup species (Appendix A), we selected 35 commonly listed chloroplast genes. To construct a whole-genome phylogenetic tree, we employed MAFFT to align the sequences and used IQ-TREE’s ModelFinder and ultrafast bootstrap (UFBoot) modules to build the tree [56,57,58,59]. To construct a CDS gene tree, 79 single-copy orthologous genes shared among the 36 species considered were extracted from their respective genomes. Each gene was aligned using MAFFT and concatenated to form a large matrix, which was then used to build a tree using IQ-TREE. For the whole-genome phylogenetic tree, the best-fit model based on the Bayesian information criterion (BIC) was K3Pu+F+I+I+R3, and the best-fit model of the CDS tree was TVM+F+R3. The whole-genome tree and CDS tree were visualized using FigTree [60].

## 4. Conclusions

Our comprehensive study of *A. edgeworthii* revealed a plastome with a genome size of 148,650 bp with 128 genes. The plastome is characterized by a predominance of palindromic repeats and a notable presence of 79 microsatellites, mainly in the LSC and SSC regions. Comparative genomic analysis across seven Glycininae species highlighted a universally conserved plastome structure and significant nucleotide diversity. Phylogenetic reconstruction of 35 Phaseoleae species emphasized *Amphicarpaea*’s affinity with Glycine, positioning *Apios* as a sister lineage to other Phaseoleae species, excluding the *Clitorinae* and *Diocleinae* subtribes. Within the Glycininae subtribe, *Amphicarpaea* and *Glycine* have a sister relationship, with *Pueraria* closely related. Our findings suggest the retention of Kennediinae within the Phaseoleae tribe and advise against the independent categorization of Erythrininae. While refining the phylogenetic contours of the Phaseoleae tribe, our findings point to the need to reevaluate the current classification of the Erythrineae. This study not only enhances our understanding of the plastomic architecture and phylogenetic relationships in the Phaseoleae tribe but also lays a foundation for future research in legume evolution and crop improvement.

## Figures and Tables

**Table 1:** Nucleotide composition in the *A. edgeworthii* plastome.

Region	A (%)	T (%)	G (%)	C (%)	GC (%)	Total (bp)	Proportion in Genome (%)
Genome	32.3	32.3	17.8	17.6	35.4	148,650	100
CDS	31.7	32.3	19.3	16.7	36.0	75,693	50.92
tRNA	34.0	32.6	18.4	15.2	33.5	22,185	14.92
rRNA	21.6	24.9	29.9	23.6	53.6	2543	1.71
Cis-spliced intron	26.2	18.9	31.5	23.5	54.9	9060	6.09
Non-coding region	35.1	34.7	15.1	15.1	30.2	49,351	33.20
LSC	33.6	33.5	16.8	16.0	32.9	83,675	56.29
SSC	35.5	35.8	13.5	15.2	28.7	17,967	12.10
IRA	28.6	29.1	21.9	20.4	42.3	23,504	15.82
IRB	28.6	29.1	21.9	20.4	42.3	23,504	15.82

**Table 2:** Genes present in the plastome of *A. edgeworthii*.

Category	Group of Genes	Name of Genes
RNA genes	Ribosomal RNA genes (rRNA)	*rrn5* ^a^, *rrn4.5* ^a^, *rrn16* ^a^, *rrn23* ^a^
	Transfer RNA genes (tRNA)	*trnC*-GCA, *trnD*-GUC, *trnE*-UUC, *trnF*-GAA, *trnfM*-CAU, *trnG*-GCC, *trnG*-UCC, *trnH*-GUG, *trnI*-GAU ^a^, *trnK*-UUU, *trnL*-CAA ^a^, *trnL*-UAA, *trnL*-UAG, *trnM*-CAU, *trnN*-GUU ^a^, *trnP*-UGG, *trnQ*-UUG, *trnR*-ACG ^a^, *trnR*-UCU, *trnS*-GCU, *trnS*-GGA, *trnS*-UGA, *trnT*-GGU, *trnT*-UGU, *trnV*-GAC ^a^, *trnV*-UAC, *trnW*-CCA, *trnY*-GUA, *trnA*-UGC ^a^
Ribosomal proteins	Small subunit of ribosome	*rps11*, *rps14*, *rps15*, *rps16* ^+^, *rps18*, *rps2*, *rps3*, *rps4*, *rps7* ^a^, *rps8*, *rps12* ^+,a^, *rps19*
Transcription	Large subunit of ribosome	*rpl14*, *rpl16*, *rpl2* ^+^, *rpl20*, *rpl22*, *rpl23* ^a^, *rpl32*, *rpl33*, *rpl36*
	DNA-dependent RNA polymerase	*rpoA*, *rpoB*, *rpoC1* ^+^, *rpoC2*
Protein genes Other genes	Photosystem I	*psaA*, *psaB*, *psaC*, *psaI*, *psaJ*, *pafI* ^++^, *pafII*
	Photosystem II	*psbA*, *psbB*, *psbC*, *psbD*, *psbE*, *psbF*, *psbH*, *psbI*, *psbJ*, *psbK*, *psbL*, *psbM*, *psbT*, *psbZ*
	Subunit of cytochrome	*petA*, *petB *^+^, *petD* ^+^, *petG*, *petL*, *petN*
	Subunit of ATP synthase	*atpA*, *atpB*, *atpE*, *atpF* ^+^, *atpH*, *atpI*
	Chloroplast envelope membrabe protien	*cemA*
	NADH dehydrogenase	*ndhA* ^+^, *ndhC*, *ndhD*, *ndhE*, *ndhF*, *ndhG*, *ndhH*, *ndhI*, *ndhJ*, *ndhK*
	Large subunit of Rubisco	*rbcL*
	Subunit acetyl-coA carboxylase	*accD*
	ATP-dependent protease subunit P	*clpP1* ^++^
	Maturase	*matK*
	C-type cytochrome synthesis	*ccsA*
	Component of the TIC complex	*ycf1* ^a^
	Hypothetical proteins	*ycf2* ^a^
	Translation initiation factor	*infA*
	N-terminal nucleophile aminohydrolases (Ntn hydrolases) superfamily protein	*pbf1*

^a^ Duplicated genes, ^+^ genes with one intron, and ^++^ genes with two introns.

**Table 3:** Genes with introns in the *A. edgeworthii* plastome and length of introns and exons.

Gene	Location	Exon I (bp)	Intron I (bp)	Exon II (bp)	Intron II (bp)	Exon III (bp)
ndhA	SSC	551	1273	541		
trnA-UGC	IR	38	807	35		
trnI-GAU	IR	42	944	37		
rps12	IR	114	535	232		
rpl2	IR	391	719	434		
rpl16	LSC	9	1176	399		
petD	LSC	8	729	475		
petB	LSC	6	812	642		
clpP1	LSC	71	708	292	790	228
rps16	LSC	40	879	230		

## Data Availability

The chloroplast genome of *A. edgeworthii* is available in the NCBI under accession number OP749930. The associated BioProject, SRA, and Bio-Sample numbers are PRJNA732525, SRR14638108, and SAMN19321915, respectively.

## Appendix A

A list of 36 plastomes of Leguminosae, all sourced from the NCBI database (https://www.ncbi.nlm.nih.gov/). This collection includes various species in the Phaseoleae tribe, which is part of the NPAAA clade: *Glycine max*, NC_007942.1; *Phaseolus vulgaris*, NC_009259; *Vigna radiata*, NC_013843.1; *Vigna angularis*, NC_021091.1; *Glycine canescens*, NC_021647.1; *Apios americana*, NC_025909.1; *Pachyrhizus eros*, NC_026682.1; *Cajanus cajan*, NC_031429.1; *Canavalia cathartica*, NC_047311.1; *Clitoria ternate*, NC_047365.1; *Butea monosperma*, NC_047384.1; *Spatholobus suberectus*, NC_048966.1; *Spatholobus pulcher*, NC_049094.1; *Canavalia gladiata*, NC_050951.1; *Lablab purpureus*, NC_054310.1; *Cajanus crassus*, NC_057277.1; *Centrosema pubes*, NC_057278.1; *Decorsea schlechteri*, NC_057444.1; *Dolichos falciformis*, NC_057447.1; *Dunbaria nivea*, NC_057448.1; *Eriosema crinitum*, NC_057449.1; *Erythrina crista*, NC_057450.1; *Fagelia bituminosa*, NC_057451.1; *Hardenbergia violacea*, NC_057453.1; *Kennedia prostrata*, NC_057454.1; *Pueraria montana*, NC_060608.1; *Amphicarpaea ferruginea*, NC_063696.1; *Pueraria edulis*, NC_065692.1; *Flemingia prostrata*, NC_065863.1; *Periandra mediterranea*, NC_067529.1; *Clitoria mariana*, NC_067531.1; *Erythrina herbacea*, NC_067539.1; *Macroptilium erythroloma*, NC_067541.1; and *Phaseolus acutifolius*, NC_067543.1 and *Amphicarpaea edgeworthii*, OP749930. The species *Minosa pudica* (NC_042921.1) was used as an outgroup.
